# Predictors of Family Strength: The Integrated Spiritual-Religious/Resilient Perspective for Understanding the Healthy/Strong Family

**Published:** 2013

**Authors:** Majid Ghaffari, Maryam Fatehizade, Ahmad Ahmadi, Vahid Ghasemi, Iran Baghban

**Affiliations:** 1 PhD Candidate, Department of Counseling, School of Psychology and Education Sciences, University of Isfahan, Isfahan, Iran.; 2 Associate Professor, Department of Counseling, School of Psychology and Education Sciences, University of Isfahan, Isfahan, Iran.; 3 Professor, Department of Counseling, School of Psychology and Education Sciences, University of Isfahan, Isfahan, Iran.; 4 Associate Professor, Department of Social Sciences, School of Literature, University of Isfahan, Isfahan, Iran

**Keywords:** Family Protective Factors, Family Strength, Spiritual Well-being

## Abstract

**Objective:** The present study aimed to investigate the effects of spiritual well-being and family protective factors on the family strength in a propositional structural model.

**Methods:** The research population consisted of all the married people of the Isfahan, Iran, in 2012 with preschool-aged children and in the first decade of marriage with at least eight grades of educational level. Three hundred and ninety five voluntary and unpaid participants were selected randomly through multi-stage sampling from seven regions of the city. The instruments used were the Spiritual Well-being Scale, Inventory of Family Protective Factors, and Family Strength Scale. Descriptive statistics and a structural equation modeling analytic approach were used.

**Results: **The analytic model predicted 82% of the variance of the family strength. The total effect of the spiritual well-being on the family strength was higher compared to the family protective factors. Furthermore, spiritual well-being predicted 43% of the distribution of the family protective factors and had indirect effect on the family strength through the family protective factors (p < 0.001).

**Conclusions:** The results of this study confirmed the interrelationships among spiritual well-being and family protective factors, and their simultaneous effects on family strength. Family counselors may employ an integrated spiritual-religious/resilient perspective to inform their strength-based work with individuals and their families.

**Declaration of interest:** None.

## Introduction

Much of the research concerning families has focused on understanding the dysfunction of families (1-3). In contrast, a small amount of research has been conducted over the past three decades with the intent of identifying what makes families “strong” or healthy (4-6). These strength-based researchers aimed to understand what makes a family strong, and tried to figure out what characteristics strong families have in common. Previous research (3) identified characteristics that seemed to be present in strong families, both in the United States and other countries. 

The model of family strengths (7) has become the model often used by family therapists, social workers, psychiatrists, and family life educators (8). Schumm et al. (9, 10) hypothesized a multivariate model of family strengths within the family, and developed a new 20-item survey designed to assess the family strength characteristics that had been embraced in a number of helpful fields. The survey assessed the family strengths of time being together, positive interaction/appreciation, open and empathetic communication/conflict resolution, commitment, and personal worth of self and others.

Moreover, as discussed by some researchers (2, 7), spiritual/religious aspects of lifestyle are an important element of family strength. Research suggested the positive impact of religious and spiritual variables are often associated with positive outcomes in individuals and families (11). Previous research (1) found that a strong family creates an atmosphere which is provided for the spiritual needs of its members by a shared set of beliefs and spiritual or religious values. These families also provided a safe environment for sharing doubts and concerns about religious beliefs (1). Strong families have a spiritual lifestyle and these families said they had an awareness of God or a Higher Power that gave them a sense of purpose and gave their family support and strength (3). 

A recently proposed comprehensive measure of one’s spirituality is “spiritual well-being” (SWB). According to Moberg and Brusek (12), SWB consists of two dimensions which seem to be a comprehensive conceptualization of spirituality. The first dimension i.e. “religious well-being” is associated with one’s relationship with a Higher Power within a particular system of religious beliefs, and the second dimension i.e. “existential well-being” is one’s sense of meaning and purpose in life. Within this definition, meaning and purpose in life is not dependent on a specific religious framework. In order to measure SWB, the Spiritual Well-Being Scale (SWBS) (13) was developed.

On the other hand, based on previous researches (1, 7, 14), strong families are also having ability to cope, adjust, change and deal with problems in a positive way. These features are similar to the term known as “family resiliency”. McCubbin et al. (15-17) initially developed and researched what has become known as The Resiliency Model of Family Stress, Adjustment, and Adaptation, which has directed the attention of helping professionals toward critical elements of family functioning from a resilience perspective. 

The Family Adaptation Model (18, 19) directly emanates from this work; however, unlike the McCubbin et al. model, there is only one simple iterative process of family adaptation rather than two processes that represent protective processes and vulnerability processes separately (20). 

Given the potential complexity of family assessment and intervention, this singular, ongoing process eliminates the tendency to dichotomize family strengths and deficits and promotes a systemic orientation that highlights reciprocity as well as parsimony and practical utility (18). The Family Adaptation Model asserts that the mediating dynamic between protective and vulnerability family processes is represented within its five dimensions: demands, appraisals, supports, coping, and adaptation (20). 

Demands represent stressors families encounter. Appraisals, social supports, and coping strategies represent the protective family processes that interact with demands or stressors to predict family adaptation (18, 19). The Inventory of Family Protective Factors (IFPF) was developed as a brief measure to assess the degree of demands or stressors and protective family factors (i.e., family resilience) perceived to be present in an individual’s family milieu, thus predicting the adaptation process (20). 

The descriptor “protective” in this context implies family members who experience higher levels of protective factors (and lower levels of stressors) in their family milieu and are less affected and thus more able to move toward adaptation when interacting with demands or stressors they encounter (i.e., protected), thereby predicting greater likelihood of “good adaptation” (21). 

Supports for the factors that are included in the IFPF are present in separate bodies of literature that represent each of them. The presence of fewer stressors in a family’s current milieu (as compared to recent and/or distant past circumstances) is in a sense “protective’. Families experiencing fewer stressors rather than more stressors or demand factors will have members less likely to develop psychological problems (22-24) and more likely to exist at an optimal level of functioning and adaptation (25, 26). 

Adaptive appraisal is defined as a family’s experience of a set of beliefs that include high self-esteem, optimism, creativity, and resourcefulness (20). Adaptive appraisal is an asset for families in increasing the likelihood of adaptively addressing problems in life, due to the fact that such appraisals serve as markers of optimal well-being; the overall balance of people’s positive and negative appraisals has been shown to predict their judgments of subjective well-being (27, 28). 

Previous studies (23, 29, 30) have addressed the role of social support and how it relates directly to psychological health. Availability of social support has been linked to emotional well-being and the ability to compensate for negative life conditions (31). Compensating experiences have been referred to as rewarding experiences that provide a sense of meaning and control over one’s life (32). Compensating experiences represent a manner of problem solving that is a cognitive enterprise with a behavioral component: “actions that help” (33).

 Clarifying issues and redefining a situation is a critical component of family coping (34). Previous research (35) posited the influence of family mastery resources as compensatory. Another study (36) likewise asserted a family’s sense of mastery to be a compensating psychological resource, a way to reduce emotional distress. 

Thus far no researcher has been done to hypothesize a unified multivariate theoretical model of the structural relationships among spiritual well-being, family protective factors (FPF), and family strengths (FS) and to test such a model. If a multivariate model of the interaction of these variables were developed, it could be helpful in a number of ways. Family life educators working with families will have a way of knowing which variables among spirituality and protective factors are the most important for a family to develop first to achieving higher strength. In order to prevent the lifelong detrimental effects of divorce and the breakdown of the family system, educators, therapists, and families must become more knowledgeable about the role that the spiritual/religious and protective factors play in preserving family strength. 

**Figure 1:**
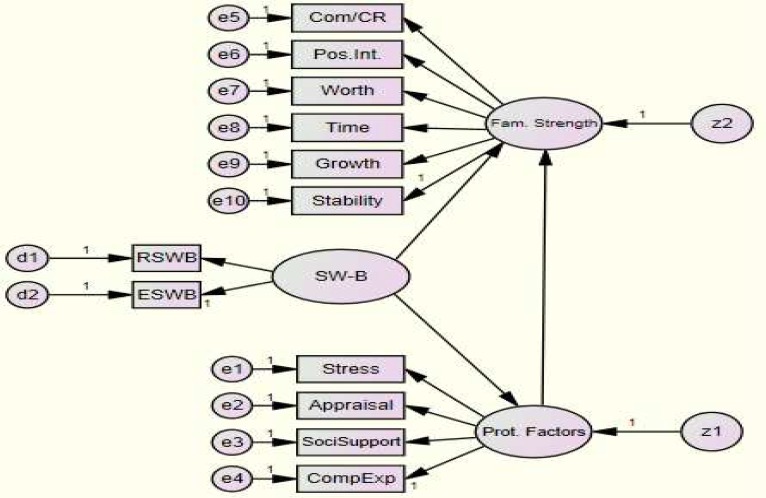
Conceptual model of the relationships among spiritual well-being (SWB), family protective  factors (FPF) and family strength (FS)

According to the literature, figure 1 was designed for conceptualizing the relationships among SWB, FPF, and FS through the Structural Equation Modeling (SEM) analytic approach.

## Materials and Methods

*Translation*

The SWBS, IFPF, and FSS were translated to the Persian in parallel by two independent, native Iranian psychology professional translators, fluent in both English and Persian. Subsequently, two translators compared the translated version and original English version of the questionnaires. Pre-testing was completed with 30 subjects to evaluate the comprehension and readability of the questionnaires. The subjects were asked whether they encountered any difficulty in understanding each of the items. The subjects indicated they had no problems with the measures and understood the items. The content validity of the translated versions was confirmed by five psychology faculty members.

*Design*

The aim of this descriptive-correlation study was to investigate the effects of SWB and FPF on the FS in a propositional structural model. Data were analyzed using SPSS for Windows 16.0 (SPSS Inc., Chicago, IL, USA) and Software Amos^™^ version 18.0. For the description of data, mean and standard deviation (SD) were used. 

Psychometric properties of instruments [alpha, test-retest reliability coefficients, and Confirmatory Factor Analysis (CFA)] were calculated. Eventually, the fit indices of the conceptual model were investigated through estimating the chi-square test (*χ*^2^), Goodness-of-Fit Index (GFI), Adjusted Goodness of Fit Index (AGFI), Comparative fit Index (CFI), Tucker-Lewis Index (TLI) and the Root Mean Square Error of Approximation (RMSEA). Values close to 1 for GFI, AGFI, CFI, and TLI were considered to be indicative of a reasonably well-fitting model and RMSEA values of close to 0.05 were considered acceptable (37).

*Sample*

The research population consisted of all the young married people of the Isfahan, Iran, in 2012 with preschool aged children and in the first decade of marriage with at least eight grades of educational level. Five hundred people were selected randomly through multi-stage sampling from the seven of the whole fourteen geographic regions of the city. The rules of privacy of the subjects’ answers were confirmed in the questionnaire instruction. After the primary investigations, 105 subjects (21%) were removed from the study sample because of their incomplete answer sheets. 

The research sample consisted of 395 (122 men and 273 women) married people with preschool aged children [range 1-3 years, mean = 0.98 (SD = 0.67) years] and in the first decade of marriage [range 1-10 years, mean length = 6 (SD = 3.46) years] at the age range of 25-35 years [mean age = 29.1 (SD = 4.44) years] with at least 8 grades of educational level (ranges of high school-MD/PhD, 21% high school, 19% diploma, 44% BA/BS, 8% MA/MS, 8% PhD/MD).

***Instruments***

*The Spiritual Well-Being Scale (SWBS)*

The 20-item SWBS (13) was developed to serve as a global psychological measure of one’s perception of SWB. The scale consists of two scales, the Religious Well-Being Scale (RWBS) (10 items), and the Existential Well-Being Scale (EWBS) (10 items). The RWB subscale assesses how one perceives the well-being of his/her spiritual life in relation to God. The EWB subscale is considered the social psychological dimension and assesses how well an individual is adjusted to self, community, and surroundings. Items are rated on a six-point Likert scale from “strongly agree” to “strongly disagree”. 

Responses to items 1, 2, 6, 9, 12, 16, and 18, were reversely scored. Therefore scores could range from 20 to 120 with higher scores indicating higher levels of SWB. Three possible scores, including the RWB subscale, the EWB subscale, and the total SWBS, were derived from item responses. 

It has been reported that the reliability coefficients for both the EWB and RWB subscales were high including test-retest reliability coefficients ranging from 0.82 to 0.99, with the exception of one sample in which a coefficient of 0.73 was observed for the EWBS. The test-retest intervals ranged from 1 to 10 weeks which was sufficient for this type of construct. 

Coefficient alphas from seven studies indicated that the internal consistency ranged from 0.72 to 0.82 for the RWB and 0.82 to 0.94 for the EWB which was satisfactory. Concurrent validity studies have been conducted to confirm that the SWBS was a direct general measure of SWB. The items on the SWBS also rendered great face validity which was determined by examination of the content of the items (13). 

In this study, the concurrent validity of translated version of SWBS was obtained by correlating the score of this questionnaire with the Daily Spiritual Experience Scale (38) (r = 0.63). Besides, the reliability coefficients of SWBS (α = 0.87 for SWBS; EWB = 0.84, RWB = 0.84, test-retest after 5 weeks = 0.81), and the fit indices from CFA on the SWBS factors (*χ*^2^ = 141.1; df = 53; GFI = 0.93; AGFI = 0.90; CFI = 0.93; RMSEA = 0.06) were satisfactory.

*The Family Strengths Scale (FSS)*

This 20-item survey assessed the family strengths of time together, positive interaction/appreciation, open and empathetic communication/conflict resolution, commitment to the growth, commitment to stability, and personal worth of self and others (9, 10). 

Items were rated on a five-point Likert scale from “strongly agree” to “strongly disagree”. Responses to items 8, 13, 15, 19, and 20, were reversely scored. Therefore scores could range from 20 to 100 with higher scores indicating higher levels of FS**. **

A former study reported the Cronbach’s alpha reliability estimates very good (> 0.80) for most subscales and more than 0.70 for all subscales (10). 

In this study, the reliability coefficients of FSS (α = 0.91 for FSS; worth = 0.70, commitment to the relationship growth = 0.83, commitment to the relationship stability = 0.60, communication/conflict resolution = 0.87, positive interaction/appreciation = 0.82, time together = 0.68, and test-retest after 5 weeks = 0.81), and the fit indices from CFA on the six factors of FSS (*χ*^2^ = 336.8; df = 151; GFI = 0.90, AGFI = 0.89; CFI = 0.93; RMSEA = 0.06) were satisfactory.

*The Inventory of Family Protective Factors (IFPF)*

The 16-item IFPF was developed as a brief measure to assess the degree of demands or stressors and protective family factors perceived to be present in an individual’s family milieu, which have satisfactory psychometric properties [Cronbach’s alpha reliability coefficient ranged 0.77 to 0.81 for all sub-scales] (20). Each of the 16 total items was written to be scored using a 5-point Likert scale. The five response options included (5) almost always, (4) generally, (3) sometimes, (2) a little, and (1) not at all like my family. 

The responses to each scale’s 4 items were summed to provide scale scores. A score of 5 was a response indicating the respondent’s perception of a very high degree of the protective factor the item represents as present in their family, whereas a score of 1 was a response indicating the respondent’s perception of a very low degree of the protective factor the item represents as present in their family. Responses to item 3, representing the fewer stressors scale, were reversely scored, item 3 representing a “response check” within that scale as the item roots were highly similar. The inventory produced a total family protective factors score (with a possible high of 80 and low of l6), as well as subscale scores (with a high of 20 and low of 4) (20). In this study, the reliability coefficients of IFPF (α = 0.91 for IFPF; fewer stressors = 0.60; adaptive appraisal = 0.82; social support = 0.88; compensating experiences = 0.89), and the fit indices from CFA on the IFPF factors (*χ*^2^ = 228.3; df = 94; GFI = 0.91, AGFI = 0.89; CFI = 0.95, RMSEA = 0.06) were satisfactory.

## Results

Tables 1 and 2 show the descriptive statistics, and the matrix of the relationships among the model variables, respectively.

As shown in table 1, there were statistically significant internal associations among all variables of the model. The correlation coefficient between FPF and FS (r = 0.71) was higher than the coefficient between SWB and FS (r = 0.67). But EWB had a higher correlation coefficient with FS (r = 0.71) than RWB (0.50), adaptive appraisal (0.69), compensating experiences (0.69), social support (0.49), and fewer stressors (0.45).

**Table 1.** Mean and standard deviations of the variables

**Variables**	**Mean**	**SD**
**Existential well-being**	43.03	08.70
**Religious well-being**	48.54	08.13
**Spiritual well-being**	91.60	15.27
**Worth**	12.60	01.98
**Commitment to stability**	10.10	02.53
**Commitment to growth**	8.76	01.31
**Communication**	23.50	04.25
**Positive interaction**	07.80	01.76
**Time together**	15.27	03.16
**Family strength**	78.82	11.83
**Fewer stressors**	14.70	03.15
**Adaptive appraisal**	15.50	03.37
**Social support**	16.08	03.83
**Compensating experiences**	15.47	03.63
**Family protective factors**	61.74	11.48

With *χ*^2^ = 98.2, df = 51, AGFI = 0.93, GFI = 0.95, CFI = 0.98, RMSEA = 0.05, and TLI = 0.95, the SEM analyses on the conceptual model of the structural associations among SWB, FPF, and FS were resulted in satisfactory indices (AGFI > 0.9; GFI > 0.90; RMSEA = 0.05; CFI > 0.90; NFI > 0.95). 

**Figure 2:**
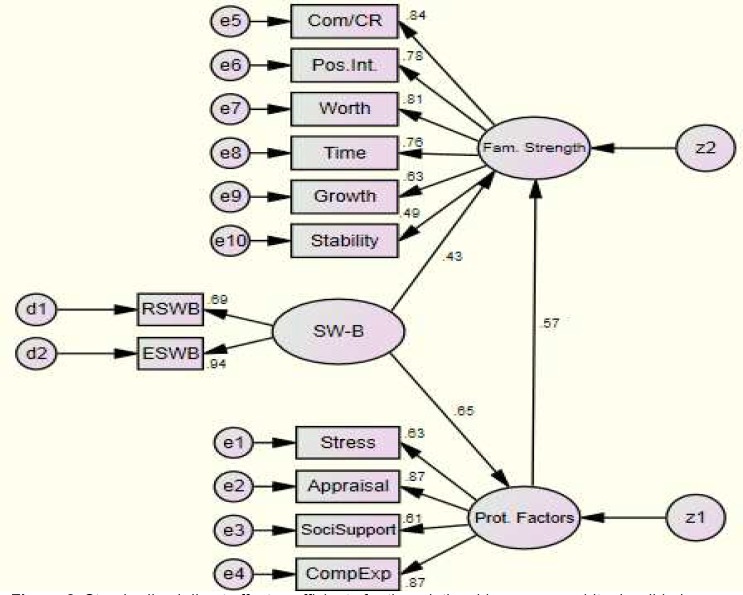
Standardized direct effect coefficients for the relationships among spiritual well-being  (SWB), family protective factors (FPF) and family strength (FS)

That is, the results showed the model fitness for conceptualizing the structural relationships among SWB, FPF, and FS. Figure 2 (analytic model), illustrates the standardized direct effect coefficients for the associations among the model variables (p < 0.001). The sample size in this study was sufficient (Hoelter’s Index > 200) (37).

As shown in Figure 2, all the effect coefficients were positive and satisfactory. The direct effect of FPF on FS (= 0.56, parameter estimate = 0.22), was higher than the direct effect of SWB (= 0.43, parameter estimate = 0. 06) on FS. In addition, SWB had strong direct effect on FPF (= 0.65, parameter estimate = 0.25). 

Table 3 shows the indirect and total effect coefficients of the model.

As shown in Table 3, the conceptual model explained 82% of the distribution of the family strength. The total standard effects of SWB (= 0.78) and FPF (= 0.56) on FS were positive and strong. Besides, the SWB predicted 43% of the distribution of the FPF and had direct total standard effect (= 0.65) on the FPF and indirect standard effect (= 0.37) on the FS through the FPF. 

The indirect and total effects of the both SWB and FPF on the communication/conflict resolution (= 0.67 and 0.48, respectively) were higher than the same effects on the worth (= 0.64 and 0.46, respectively), positive interaction/appreciation (= 0.62 and 0.44, respectively), time together (= 0.61 and 0.43, respectively), commitment to the relationship growth (= 0.50 and 0.36, respectively), and commitment to the relationship stability (= 0.39 and 0.28, respectively). Besides, the total effects of SWB on the adaptive appraisal and compensating experiences (= 0.57 for both of them) were higher than the same effects on the fewer stressors (= 0.41), and social support (= 0.40).

**Table 2:** The correlation matrix of the variables

**Variables**	**1**	**2**	**3**	**4**	**5**	**6**	**7**	**8**	**9**	**10**	**11**	**12**	**13**	**14**	**15**
**Existential well-being**	1														
**Religious well-being**	0.65**	1													
**Spiritual well-being**	0.91**	0.90**	1												
**Worth**	0.63**	0.47**	0.61**	1											
**Commitment to stability**	0.43**	0.30**	0.41**	0.32**	1										
**Commitment to growth**	0.49**	0.44**	0.51**	0.56**	0.30**	1									
**Communication/conflict resolution**	0.61**	0.44**	0.58**	0.66**	0.48**	0.53**	1								
**Positive interaction/appreciation**	0.55**	0.40**	0.53**	0.65**	0.41**	0.45**	0.64**	1							
**Time together**	0.59**	0.36**	0.53**	0.62**	0.33**	0.44**	0.64**	0.67**	1						
**Family strength**	0.71**	0.50**	0.67**	0.80**	0.62**	0.64**	0.90**	0.80**	0.81**	1					
**Fewer stressors**	0.40**	0.31**	0.39**	0.37**	0.22**	0.31**	0.41**	0.42**	0.35**	0.45**	1				
**Adaptive appraisal**	0.54**	0.41**	0.52**	0.59**	0.38**	0.45**	0.63**	0.58**	0.56**	0.69**	0.57**	1			
**Social support**	0.34**	0.24**	0.32**	0.44**	0.22**	0.38**	0.42**	0.43**	0.39**	0.49**	0.49**	0.50**	1		
**Compensating experiences**	0.52**	0.45**	0.54**	0.57**	0.31**	0.48**	0.64**	0.59**	0.59**	0.69**	0.53**	0.76**	0.53**	10.86**	1
**Family protective factors**	0.55**	0.42**	0.54**	0.60**	0.34**	0.50**	0.64**	0.62**	0.58**	0.71**	0.77**	0.86**	0.78**		

* p < 0.001

**Table 3:** The indirect and total effect coefficients of the model

**Variable**	**Indirect effect**		**Total effect**		**R** ^2^
	**Parameter estimate**	**Standardized effects**	**Parameter estimate**	**Standardized effects**	
**On the FPF**					0.43
**SWB**			0.25	0.65	
**On the FS**					0.82
**SWB**	0.06	0.37	0.12	0.78	
**FPF**			0.22	0.56	
**On the wor**					0.65
**SWB**	0.16	0.64	0.16	0.64	
**FPF**	0.29	0.46	0.29	0.46	
**On the com stab**					0.24
**SWB**	0.12	0.39	0.12	0.39	
**FPF**	0.24	0.28	0.24	0.28	
**On the com Gro**					0.63
**SWB**	0.08	0.50	0.08	0.50	
**FPF**	0.15	0.36	0.15	0.36	
**On the Com/CR**					0.71
**SWB**	0.35	0.67	0.35	0.67	
**FPF**	0.64	0.48	0.64	0.48	
**On the PI/Appr.**					0.61
**SWB**	0.13	0.62	0.13	0.62	
**FPF**	0.25	0.44	0.25	0.44	
**On the Tim**					0.40
**SWB**	0.23	0.61	0.23	0.61	
**FPF**	0.43	0.43	0.43	0.43	
**On the stress**					0.40
**SWB**	0.16	0.41	0.16	0.41	
**On the Adao Appr.**					0.76
**SWB**	0.23	0.57	0.23	0.57	
**On the So. Sup.**					0.38
**SWB**	0.19	0.40	0.19	0.40	
**On the Com Expr.**					0.76
**SWB**	0.25	0.57	0.25	0.57	

< 0.001; SWB: Spiritual well-being; FPF: Family protective factors; FS: Family strength; Wor: Worth; Com. Stab.: Commitment to stability; Com. Gro.: Commitment to growth; Com/CR: Communication/Conflict resolution; PI/Appr.: Positive interaction/appreciation; Tim.: Time together; Low. Stre. :Lower stressors; Adap. Appr.: Adaptive appraisals; So. Sup.: Social support; Com. Expr.: Compensating experiences

## Discussion

The aim of this study was to investigate the effects of SWB and FPF on the FS in a propositional structural model. As showed in Figure 2 and Table 3, a positive direct effect of SWB on FS was observed. As discussed earlier, previous studies (1, 2, 7, 8, 11) suggested that spiritual/religious aspects of lifestyle are an important element of FS, and the positive impact of religious and spiritual variables are often correlated with positive outcomes in individuals and families. 

As discussed, a strong family creates an atmosphere which provided for the spiritual needs of its members by a shared set of beliefs and spiritual or religious values. These families also provides a safe environment for sharing doubts and concerns about religious beliefs and they also have an awareness of God or a Higher Power that give them a sense of purpose and give their family support and strength and this awareness helps them to be more forgiving, more patient with each other, and to be more positive and supportive. A positive effect of religious/spiritual aspects of one’s life on family strength through enhancing person’s feeling of worth was confirmed by previous research (8). 

The results of this study also showed a positive direct effect of FPF on FS. Research reports and literature reviews over the past decade have provided family counselors with an enhanced understanding of, as well as protocol for, employing a family resilience perspective to inform their work with individuals and their families (20, 39-43). Resilience within a family context highlights families’ positive adjustment in the context of challenging life conditions (44). 

Family resilience emanates from a systemic view positing the presence of vulnerability processes and protective processes reciprocally interacting to affect the functioning of a family and all its members in a circular manner (45). Based on previous research studies (22-32, 35, 46), family protective factors are positively related to family and family members’ strength. Experiencing fewer stressors will results in having family members less likely to develop psychological problems (22-24) and more likely to exist at an optimal level of functioning and adaptation (26).

Adaptive appraisal includes family members’ beliefs that their family possesses a sense of self-efficacy, positive expectations, acceptance of life situations, and maintenance of trust and calm (18). This factor involves how a family and its members view and approach crisis situations, which subsequently influences their potential solution efforts (47). Previous research confirmed that, adaptive appraisal helps families to increase the likelihood of adaptively addressing problems in life, because such appraisals serve as markers of optimal well-being (27, 28).

Social support is defined as a family’s experience of having at least one supportive, caring, interested and/or trusting relationship (20). According to the previous research, availability of social support through providing emotional well-being and the ability to compensate for negative life conditions (31), positively affects family strength.

Compensating experiences are defined as a family’s experiences of mastery within the context of adversity (20). This mastery includes feelings of positive control over uplifting experiences, while having experienced the same situations as hassles (48). According to previous findings (32, 33), compensating experiences can be considered as a manner of problem-solving which through providing a sense of meaning and control over one’s life, positively effects family strength. 

As noted earlier, researchers (35, 36) asserted a family’s sense of mastery to be a compensating psychological resource, a way to reduce emotional distress.

In this study, results also showed a significant strong indirect effect of SWB on FS through FPF which can be explained through reviewing and integrating the results of some related previous studies. Folkman stated that research supports the distinction of meaning-based coping from other forms of coping and suggested that religious and spiritual coping is an important aspect of meaning-based coping (48). 

In Calicchia and Graham’s (49), SWB was positively correlated with health and had a negative association with stress variables. These participants reported higher levels of SWB, reported less stress from one’s spouse/partner and extended family. They also reported that according to their results, SWB was positively correlated with receiving social support from extended family, friends, and positive events. Given the findings, they concluded that SWB was an effective buffer of stress and an effective provider of social support. 

Another study (50) reported a positive effect of spirituality and social support on the family resilience. Previous research confirmed that SWB has been positively associated with positive outcome, higher quality of coping, and more adaptive appraisal in the midst of various difficult life circumstances through providing a clear sense of meaning and direction in life (49-52). 

## Conclusion

The obtained results confirmed the interrelationships between SWB and FPF, and their simultaneous effects on FS and suggested that family counselors employ an integrated spiritual-religious/resilient perspective to inform their strength-based work with individuals and their families. of course.

More studies are needed with different measures (different measures based on different conceptualizing of spirituality and religiosity) and in different populations (e.g. different socio-economic levels, different cultures and sub-cultures, investigating the conceptual model based on gender difference) to provide a comprehensive theoretical explanation for the interrelationships among spiritual/religious variables, family resilience and family strength.
